# Recent advances in understanding neuronal diversity and neural circuit complexity across different brain regions using single-cell sequencing

**DOI:** 10.3389/fncir.2023.1007755

**Published:** 2023-03-30

**Authors:** Yu Xing, Chunfang Zan, Lu Liu

**Affiliations:** ^1^Department of Neurology, Beidahuang Industry Group General Hospital, Harbin, China; ^2^Institute for Stroke and Dementia Research (ISD), LMU Klinikum, Ludwig-Maximilian-University (LMU), Munich, Germany; ^3^Munich Medical Research School (MMRS), LMU Klinikum, Ludwig-Maximilian-University (LMU), Munich, Germany

**Keywords:** single-cell sequencing, neurons, neural circuits, cortex, hypothalamus, hippocampus, synaptic connection, neuroscience

## Abstract

Neural circuits are characterized as interconnecting neuron networks connected by synapses. Some kinds of gene expression and/or functional changes of neurons and synaptic connections may result in aberrant neural circuits, which has been recognized as one crucial pathological mechanism for the onset of many neurological diseases. Gradual advances in single-cell sequencing approaches with strong technological advantages, as exemplified by high throughput and increased resolution for live cells, have enabled it to assist us in understanding neuronal diversity across diverse brain regions and further transformed our knowledge of cellular building blocks of neural circuits through revealing numerous molecular signatures. Currently published transcriptomic studies have elucidated various neuronal subpopulations as well as their distribution across prefrontal cortex, hippocampus, hypothalamus, and dorsal root ganglion, etc. Better characterization of brain region-specific circuits may shed light on new pathological mechanisms involved and assist in selecting potential targets for the prevention and treatment of specific neurological disorders based on their established roles. Given diverse neuronal populations across different brain regions, we aim to give a brief sketch of current progress in understanding neuronal diversity and neural circuit complexity according to their locations. With the special focus on the application of single-cell sequencing, we thereby summarize relevant region-specific findings. Considering the importance of spatial context and connectivity in neural circuits, we also discuss a few published results obtained by spatial transcriptomics. Taken together, these single-cell sequencing data may lay a mechanistic basis for functional identification of brain circuit components, which links their molecular signatures to anatomical regions, connectivity, morphology, and physiology. Furthermore, the comprehensive characterization of neuron subtypes, their distributions, and connectivity patterns *via* single-cell sequencing is critical for understanding neural circuit properties and how they generate region-dependent interactions in different context.

## Introduction

Neurons, the most critical structural as well as functional components of the nervous system, are divided into sensory neurons, motor neurons, and interneurons in the functional sense (Peng et al., 2021; Zeng, 2022). Due to their genetic diversity and wide distribution across brain regions, specific types of neurons generally have distinct functions (Beine et al., 2022; Breton-Provencher et al., 2022). Interestingly, these neurons do not work individually. In other words, developing neurons and their synaptic partners interconnect with each other, forming complex neural circuits to exert specific functions once activated (Sanes and Zipursky, 2020; Luo, 2021; Brinkman et al., 2022). In fact, there are a vast number of neural circuits in the brain, governing diverse functions (Hu et al., 2020; Kuner and Kuner, 2021). From a clinical perspective, aberrant neural circuits have been observed to participate in the initiation and progression of a variety of neurological disorders, such as autism spectrum disorder (ASD) and neurodegenerative diseases, through pathological neuronal activities and abnormal axon guidance protein changes (Van Battum et al., 2015; Sudarov et al., 2018; Barth and Ray, 2019; Moussa and Wester, 2022). Taking ASD as an example, an imbalance in the ratio of excitation to inhibition within cortical circuits has been hypothesized as a specific developmental mechanism (Golden et al., 2018; Sohal and Rubenstein, 2019). Therefore, it is reasonable to focus on and further reveal region-specific neuronal diversity, which supports us to better understand the complexity of neural circuits that underlie autistic phenotypes or others.

**FIGURE 1:**
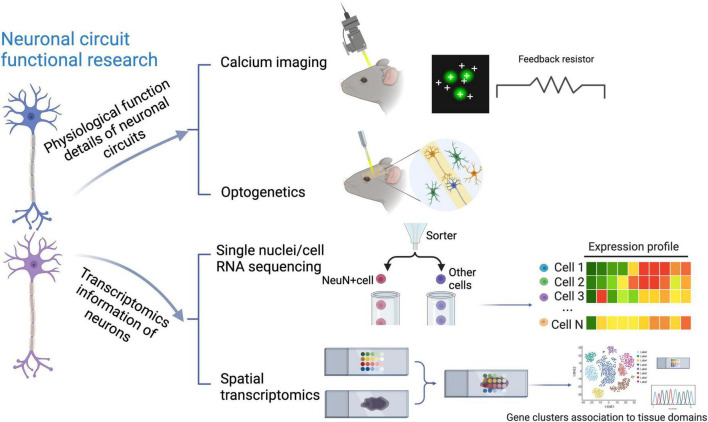
Traditional and transcriptomic techniques to study neural circuits. Depicted are the traditional techniques including optogenetics and calcium imaging, and recently-applied single-cell transcriptomic approaches, which have been used to investigate neural circuits from different aspects.

To this end, quite a lot of methods to study neural circuits have been emerging, such as optogenetics and calcium imaging, nicely linking distinct circuit abnormalities to specific disease dimensions, as shown in the above part of Figure 1. However, they do not take neuronal diversity across different brain regions into account (Bugeon et al., 2022; Lin et al., 2022; Russell et al., 2022). In contrast, recent application of multiple single-cell sequencing techniques, as detailedly compared in Table 1, through identifying various molecular signatures in about 1,000∼1,000,000 individual cells, provides high-resolution genomic information for neurons, with more details in neuronal subpopulation identification (Lange et al., 2020; Cebrian-Silla et al., 2021), although it also meets some technical challenges (Stegle et al., 2015; Stubbington et al., 2017). In terms of the assessment of neural cells, one of the limitations of single-cell sequencing is that some subtypes of neurons, for example cortical layer five pyramidal tract neurons, may not easily survive during the cell isolation process (Tasic et al., 2018). Therefore, most researchers performed their experiments on neurons *via* single nuclei RNA sequencing (snRNA-Seq) (Cameron et al., 2022; Smajić et al., 2022). On the other hand, recent progress in single-cell RNA sequencing (scRNA-seq) techniques also makes it possible to perform the transcriptional cataloging of neural cells, including neurons and astrocytes, as summarized by several nice review articles (Mu et al., 2019; Yuste et al., 2020; Armand et al., 2021). Of note, the comprehensive characterization of neuronal diversity and the precise identification of neuron-specific transcriptional features *via* snRNA-seq can extend our current understanding of neural circuits and further predict state modulations of different functional neurons. When combining regular snRNA-seq with spatial transcriptomics, such as the multiplexed error robust fluorescence *in situ* hybridization (MERFISH), as a notable tool (Moffitt et al., 2016; Burgess, 2019; Eng et al., 2019), it brings great potential to draw more complicated molecular maps. Major technical features of different single-cell sequencing approaches, including applied protocols, the number of detected genes, and sample requirements, etc., have been summarized in Table 1.

**TABLE 1:** Comparisons of different single-cell transcriptomic techniques.

	Levels	Common protocols	Number of detected genes	Cell types	Sample requirements
Single-cell RNA seq	Single cell level	10X Genomics SMART-seq2 Marseq	Up to 20,000 genes (mRNA of cytoplasm)	Neural cells except most neurons	Fresh tissue
Single-nuclei RNA seq	Single nucleus level	10X Genomics SMART-seq2	Less than the number from single-cell RNA seq (mRNA of nucleus)	All cell types	Fresh frozen, PFA-fixed frozen tissue
Single-cell transcriptome profiling technology (spatial transcriptomics)	Single cell level	MERFISH	Currently up to 500 selected genes	All cell types	Fresh frozen, PFA-fixed frozen tissue, paraffin-embedded tissue
Spatial transcriptomics	Single area	10X Genomics (the Visium Spatial platform)	Up to 20,000 genes (mRNA of cytoplasm)	All cell types	Fresh frozen, PFA-fixed frozen, formalin-fixed, paraffin-embedded tissue

**FIGURE 2:**
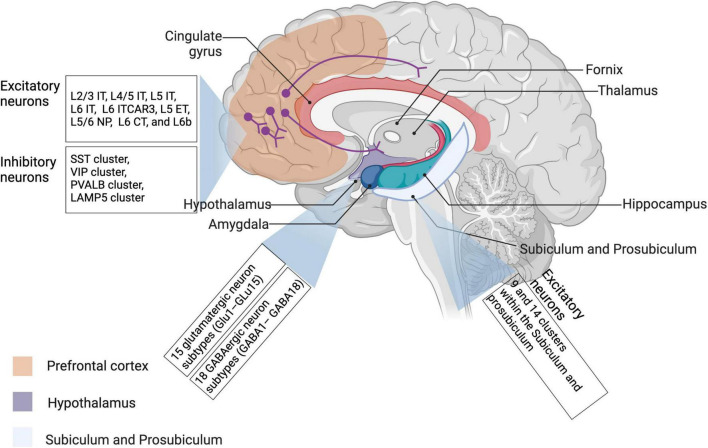
Neuronal diversity across different brain regions revealed by single-cell transcriptomic studies. Most recent literature about neuronal transcriptomes have investigated neuron subpopulations from prefrontal cortex, hippocampus, and hypothalamus. Relevant details have been also described in Table 2. IT, intratelencephalic-projecting neurons; ET, extratelencephalic-projecting neurons; NP, near-projecting neurons; CT, corticothalamic projecting neurons.

Taking current progress in understanding neuronal diversity and neural circuit complexity, as well as their potential significance in neurological diseases and psychiatric disorders, into account, we would like to make a brief summary of the latest studies identifying uncharacterized subtypes of neurons and new neural circuits by scRNA-seq in accordance with their specific distributions in the brain. In addition, common scRNA-seq techniques applied in these studies have been reviewed in Table 1. While primarily focusing on the application of scRNA-seq, we keep updates on recent single-cell transcriptomics studies showing intriguing findings about neuronal diversity and region-specific neural circuits among prefrontal cortex, subpallium, hypothalamus, hippocampus, dorsal root ganglion, and brainstem in different species including humans, mice, and birds, which have been listed in Table 2, and vividly visualized in Figure 2, and will be elaborated in the following chapters as well. Of note, some spatially resolved advances demonstrated by spatial transcriptomics will be additionally discussed here, giving new insights into how multi-regional neural circuits are organized in this context.

**TABLE 2:** Examples investigating neuronal diversity and neural circuit complexity across different regions using single-cell sequencing.

Regions	Sub-regions	Single-cell seq techniques	Species	Major neuron types	Relevant circuits	References
Cerebral Cortex	Primary motor cortex	10X Genomics SMART-seq2	Mice	Intratelencephalic neurons	Cortical circuits	Zhang et al., 2021
	Song motor regions	10X Genomics	Songbirds	Glutamatergic neurons, GABAergic neurons, LGE-class neurons	Vocal circuits	Colquitt et al., 2021
	Neocortex	SMART-seq	Mice	Glutamatergic neurons, GABAergic neurons	Neocortical circuits	Tasic et al., 2018
	Prefrontal cortex	SMART-seq2	Humans	Intermediate progenitor cells, excitatory neurons	Cortical circuits	Zhong et al., 2018
	Cortex	CEL-seq	Mice	GABAergic neurons	Cortical circuits	Paul et al., 2017
Subpallium	Fetal subpallium	10X Genomics	Humans	Interneurons	Not specified	Yu et al., 2021
Hippocampus	Subiculum, prosubiculum	SMART-seq 10X Genomics	Mice	Glutamatergic neurons	Hippocampal circuits	Ding et al., 2020
	Hippocampus	Drop-seq	Reptiles	Glutamatergic neurons, GABAergic neurons	Hippocampal circuits	Tosches et al., 2018
Diencephalon	Hypothalamus	SMART-seq2	Mice	Hypothalamic neurons	Hypothalamic circuits	Hanchate et al., 2020
	Hypothalamus	SMART-Seq2	Mice	Oxytocin neurons	Parallel processing circuits	Lewis et al., 2020
	Ventral posterior hypothalamus	10x Genomics	Mice	VPH neurons	VPH circuits	Mickelsen et al., 2020
	Hypothalamic preoptic region	Drop-seq	Mice	Hypothalamic preoptic neurons	Hypothalamic preoptic circuits	Moffitt et al., 2018
	Hypothalamus	Drop-seq	Humans	Hypothalamic neurons, non-neuronal cells	Hypothalamic circuits	Chen et al., 2017
Dorsal root ganglion	Dorsal root ganglion	SMART-seq	Mice	Somatosensory neurons	Sensory circuits	Li et al., 2016
	Dorsal root ganglion	Single-cell RNA sequencing (undefined)	Mice	Sensory neurons	Somatic sensation	Usoskin et al., 2015
	Dorsal root ganglion	Single-cell RNA sequencing (undefined)	Mice	Sensory neurons	Sensory circuits	Chiu et al., 2014
Brainstem (ventral tegmental area)	Substantia nigra pars compacta	10X Genomics (snRNA-seq)	Humans	Dopamine neurons	Not specified	Kamath et al., 2022
Retina	Retina	Single-cell RNA sequencing (undefined)	Mice	Bipolar neurons	Core regulatory circuits	Norrie et al., 2019
Cerebellum	Cerebellum	Droplet-based RNA-seq	Mice	Interneurons	Cerebellar circuit	Peng et al., 2019
Spinal cord	Spinal cord	SPLiT-seq	Mice	Glutamatergic neuron, GABAergic neurons, interneurons	Not specified	Rosenberg et al., 2018

## Potential value of single-cell sequencing in studying neuronal diversity and neural circuit complexity

Beyond different regions in the brain and among several species, individual neurons generally process information through forming neuronal circuits with their synaptic partners, involving precise synaptic connectivity. Of note, the evolution and development of neuronal circuit architectures have been comprehensively reviewed by Luo (2021) recently, at least partially showing how different architectures of neuronal circuits cooperate in an individual nervous system. In addition, identifying new subpopulations of region-specific neurons and even their molecular signatures is of importance to a deeper understanding of neural circuits underlying abnormal behaviors, as exemplified by the identification of Glu*^LHA^* neurons in a neural circuit for the inhibition of feeding under persistent pain (Tang et al., 2022). In this review, a wide range of applications of traditional techniques, including optogenetics and calcium imaging *versus* currently applied single-cell sequencing approaches have been visualized in Figure 1. Interestingly, optogenetics and calcium imaging using two-photon microscopy give some insights into physiological functions of neuronal circuits by detecting the spiking activity of neuronal populations, whereas single-cell sequencing techniques additionally provide the transcriptomic information for neurons at the single-cell resolution (Quirin et al., 2016; Armand et al., 2021; Rodriguez-Romaguera et al., 2022). These three techniques complement each other to elucidate neural circuits (Figure 1). Recently, several comparative studies encompassing different species have suggested that investigating various subtypes of neurons in homologous brain regions *via* single-cell transcriptomics is a useful first step (Tosches et al., 2018; Hodge et al., 2019; Kebschull et al., 2020). Afterward, with the development of single-cell transcriptomics in the past decade, plenty of original studies revealing region-specific neuron subpopulations and related neural circuits have been emerging, as detailedly summarized in Table 2.

So far, there are more than 20 kinds of established protocols for single-cell sequencing since this technique was first introduced by Tang et al. (2009). Transcriptomic data generated by different protocols display significant heterogeneity (Ziegenhain et al., 2017; Hwang et al., 2018; Mereu et al., 2020). For this, Mereu et al. (2020) have systematically compared thirteen kinds of commonly used protocols of scRNA-seq and snRNA-seq applied to a heterogeneous reference sample resource and discussed their major differences with regards to the RNA capture efficiency, bias, scale and costs, comprehensiveness, and integrability. Of interest, most of the improved versions of plate-based methods, such as Quartz-seq2, CEL-seq2 and Smart-seq2, have been found to generate high-resolution transcriptome profiles. As shown in current studies, Smart-seq/Smart-seq2 and 10X Genomics, as the most representative sequencing protocols, have been extensively applied to reveal genetic gene-expression heterogeneity of neural cells (Hanchate et al., 2020; Maynard et al., 2021; Wang et al., 2021). These protocols also hold true for the identification of diverse neuron subtypes and new neural circuits in humans (Zhong et al., 2018; Yu et al., 2021; Kamath et al., 2022), and mice (Ding et al., 2020; Hanchate et al., 2020; Zhang et al., 2021), details of which have been summarized in Table 2. Additionally, this review also includes a few studies utilizing other sequencing protocols, such as CEL-seq and Drop-seq (Chen et al., 2017; Paul et al., 2017).

It is worth noting that even though sc/snRNA-seq assists in characterizing the transcriptomes of rare types of cells and accurately understanding gene expression regulatory mechanisms, one of the most striking disadvantages drawing our attention is the sacrifice of the spatial context in neural circuits due to the disruption of tissues into isolated cells (Kolodziejczyk et al., 2015; Armand et al., 2021). To tackle this problem, spatial transcriptomics have emerged as a collection of genomic technologies, which can dissect neural circuits with complex anatomical organization, to provide comprehensive anatomical and functional information with spatial localization messages (Lein et al., 2017; Moffitt et al., 2018; Kim et al., 2019). However, analyses of spatial transcriptomic data are computationally challenging (Wang et al., 2018; Shang and Zhou, 2022). Nevertheless, spatially resolved transcriptomics of brain tissue retain the spatial context at the regional, cellular or sub-cellular levels, providing more gene expression information for clustering single-neuron populations. Interestingly, Shah et al. (2016, 2017) have applied sequential fluorescence *in situ* hybridization (seqFISH) using mouse brain tissues to detect the gene expression in single cells within a large dynamic range, profiling a complex molecular map of hippocampal neurons with high spatial heterogeneity. However, spatial transcriptomics have some technical limitations as well, in comparison with conventional single-cell sequencing methods, as exemplified by the predefinition of candidate genes, the complicated experimental setup, high requirements for computational images, and high cost, and the time-consuming imaging, which all limit their applications, and further ask researchers for more experimental inputs (Rodriques et al., 2019; Armand et al., 2021).

Even so, spatial transcriptomics, as an intriguing and emerging technique, geniusly integrate cellular transcriptomics with their spatial coordinates within tissues, together allowing a deeper understanding of cellular composition, and heterogeneity as well as cell-cell communications (Wolock et al., 2019; Anderson et al., 2022; Fang S. et al., 2022; Shen et al., 2022). On the other hand, neuronal activities in local neural circuits have been thought to be organized for information processing both spatially and temporally, which can be at least partially explained by spatial transcriptomics and other structural analyses (Weisenburger and Vaziri, 2018; Parra-Damas and Saura, 2020; Endo et al., 2021). In addition, given specific cognitive functions as well as distinct computational properties of different brain areas, we would like to discuss these single-cell transcriptomic studies addressing neuronal diversity and neural circuit complexity according to the distribution of neurons in the brain in the following chapters, respectively.

## Advances in studying cortical neurons and circuits *via* single-cell sequencing

Due to the size and the intricate folding, the cerebral cortex takes a predominant place in the brain among species (Fernández et al., 2016; Llinares-Benadero and Borrell, 2019; Bhattacharjee et al., 2021). Thus, the cortex remains the most well-studied brain region through single-cell sequencing so far, attracting a lot of attention from researchers. Lodato et al. (2015) have profiled various molecular signatures of 36 neurons from the cerebral cortex of three healthy individuals by single-cell sequencing and identified thousands of somatic single-nucleotide variants. Furthermore, Heavner et al. (2020) have defined many subclasses of developing projection neurons in the cerebral cortex according to the transcription factor expression, which is in line with single-cell RNA-seq subtypes, as confirmed through multidimensional approaches. In this chapter, involved cortical neurons associated with neural circuit formation and changes mainly include intratelencephalic neurons, glutamatergic neurons, GABAergic neurons, LGE-class neurons, and intermediate progenitor cells (Paul et al., 2017; Tasic et al., 2018; Zhong et al., 2018; Colquitt et al., 2021; Zhang et al., 2021). More recently, Endo and coworkers have comprehensively reviewed the emerging technologies for studying local neural circuits in the cerebral cortex and given new insight into local neural circuits obtained by these technologies, such as single-cell sequencing and tissue clearing, etc. (Endo et al., 2021). In this review, we mainly focus on the application of single-cell sequencing in studying neuronal diversity and neural circuit complexity. For this, relevant details of these original findings, including applied single-cell sequencing techniques, major studied species, and involved brain regions, have been systemically summarized in Table 2, and visualized in Figure 2 as well.

From a conventional hierarchical view of cortical circuits, neurons are regarded as specialized structures in response to specific stimuli, process these signals, and transmit this information to neurons in the following hierarchical order. These cortical neurons collect information that they receive from other circuits and encode a percept (Solari et al., 2019; Brinkman et al., 2022). However, Zhou et al. (2018) have suggested this hierarchical view of visual cortical processing may not apply to the mouse visual cortex, as tested in their study. What single-cell sequencing can add to this view is to extend the understanding of the diversity of neuronal types in the brain and give more molecular details for neuron-neuron communications. To this end, significant diversity in excitatory and inhibitory neurons in the cerebral cortex revealed by single-cell sequencing will be discussed. Glutamatergic neurons, as the most common and widely studied excitatory neurons in cortical circuits, have important molecular signatures and specific physiological properties (Birey et al., 2017; Chapman et al., 2022; Zhong et al., 2022). This importance also holds true for GABAergic neurons in inhibitory circuits and long-range projections (Rock et al., 2017; Kirmse and Zhang, 2022). Several transcriptomic studies have consistently demonstrated that glutamatergic neurons show a greater diversity across brain regions and species in comparison with GABAergic neurons (Tasic et al., 2018; Bakken et al., 2021; Yao et al., 2021), which is in line with single-cell DNA methylation data from Luo et al. (2017).

Through utilizing SMART-seq, Tasic and coworkers have identified some new types of glutamatergic and GABAergic neurons in mouse neocortex, which is responsible for coordination of learned behaviors. Of interest, some markers used for cell type assignment are novel, such as *Slc30a3*, *Osr1*, and *Fam84b*, etc. In their study, glutamatergic neurons display a region-specific pattern, whereas most of GABAergic neurons are ubiquitously located across two neocortical areas, i.e., the primary visual cortex, and the anterior lateral motor cortex. Specifically, glutamatergic neurons from two areas whereas belonging to the same cluster have a median of 78 differentially expressed genes (DEGs), whereas GABAergic types only have a median of two DEGs, as demonstrated by the best-matched tests (Tasic et al., 2018). In a similar vein, Paul et al. (2017) have applied CEL-seq to characterize murine GABAergic subpopulations in mouse cerebral cortex, which are distinguished by their transcriptional architectures. These identified gene families can be divided into 6 functional categories, including transcription factors, cell-adhesion molecules, regulatory components of membrane-proximal signaling pathways, ion channels, neurotransmitter and modulator receptors, and neuropeptides and vesicular release components. In addition, some special features of GABA have been found to associate with distinct spatiotemporal patterns of receptor activation and post-synaptic cell firing that affect circuit computation (Markram et al., 2015). Paul et al. (2017) have also revealed 190 kinds of DEGs, and confirmed some known markers for medial ganglionic eminence- and caudal ganglionic eminence-derived interneurons, which may link the altered gene expression to aberrant cellular and circuit properties. It is worth noting that there exists a huge difference in the number of DEGs between the above-mentioned two studies from Tasic et al. (2018), Paul et al. (2017), which could be due to different protocols of single-cell sequencing applied in their experiments.

Zhong et al. (2018) have revealed molecular signatures of neural progenitor cells, excitatory neurons, and interneurons, etc., through analyzing more than 2,300 single cells in human prefrontal cortex that is associated with advanced cognitive functions, and neurogenesis dynamics during neuronal differentiation (Zhou et al., 2018; Lui et al., 2021). In turn, dysfunction of the prefrontal cortex may lead to cognitive deficits and most of neurodevelopmental disorders (Butt and Lak, 2020; Anderson et al., 2021). As addressed in the above part, given their origin across the dorsal telencephalon, transcriptomic profiles of cortical excitatory neurons may show a more heterogeneous pattern compared with GABAergic neurons (Mayer et al., 2018). Therefore, these transcriptomic findings together outline general characteristics of different neuronal populations that may actively participate in cortical circuits. Interestingly enough, Colquitt et al. (2021) have studied another species except for humans and mice, birds, using 10X Genomics, and demonstrated that glutamatergic vocal neurons of birds are quite similar to neocortical projection neurons of mammals concerning their transcriptional activities. As shown in Table 2, glutamatergic neurons have been especially investigated in mouse hippocampus using 10X Genomics, which will be discussed in the next paragraph (Tasic et al., 2018; Ding et al., 2020). More recently, Zhang and coworkers have applied both 10X Genomics and SMART-seq2, together with MERFISH to establish a spatially resolved cell atlas, with special emphasis on intratelencephalic neurons. In their study, 95 neuronal and non-neuronal cell clusters have been profiled in the murine primary motor cortex, and further a comprehensive spatial map of excitatory as well as inhibitory neuronal clusters has been revealed based on the application of MERFISH (Zhang et al., 2021).

Moreover, single-cell spatial transcriptomics together with retrograde labeling can provide the laminar and regional location of neurons with specific projections. Notably, the integration of MERFISH in their experiment shows a complex network among multiple neuronal clusters beyond brain regions (Xia et al., 2019; Zhang et al., 2020). Even though there are a limited number of spatial transcriptomic studies on neural circuits, MERFISH seems to be able to catch the spatial complexity of neuronal circuits *via* collecting various forms of spatial data from the same region in the brain. Of note, cell type-specific inference of differential expression (C-SIDE), a novel statistical method, supports to identify cell type-specific differential expression in spatial transcriptomics, providing additional possibilities for mechanistic exploration of diverse circuits (Cable et al., 2022). Furthermore, Fang R. et al. (2022) have utilized MERFISH to reveal differences in somatic interactions as well as conservation in the laminar organization of cells between mice and humans, suggesting potential commonalities and features in neural circuits across species

At last, in addition to neuronal development, single-cell sequencing could evaluate gene expression changes induced by neural activity and plasticity, as exemplified by visual cortical neurons exposed to light (Hrvatin et al., 2018; Wei et al., 2022). In turn, the analysis of activity-regulated genes is also helpful to identify active cells *via* single-nucleus RNA-seq (Hu et al., 2017). These findings together suggest that single-cell sequencing not only reveals neuron diversity to explore putative functions, but also assesses DEGs to analyze context-specific neurons. It is worthy to mention here that more researchers prefer to use multiple single-cell sequencing techniques to strengthen the credibility of those findings nowadays (Ding et al., 2020; Zhang et al., 2021). In summary, categorizing cortical neurons into specific subtypes by single-cell sequencing techniques, and investigating the roles of different types of neurons in the function of the circuit, is an essential step toward understanding how different cortical circuits produce distinct computations.

## Advances in studying hypothalamic neurons and circuits *via* single-cell sequencing

In addition to the cerebral cortex addressed in the above part, the diencephalon, including the hypothalamus, is the second most well-studied brain region by researchers using single-cell sequencing (Chen et al., 2017; Ding et al., 2020; Hanchate et al., 2020). In general, hypothalamic neurons are highly diverse and participate in a wide range of processes and behaviors which are essential for organismal survival (Sternson, 2013; Li et al., 2018; Fu et al., 2019). On the one hand, both hypothalamus and hippocampus belong to the limbic system, which is in charge of advanced mental functions, such as emotion processing and time perception (Gley et al., 2021). On the other hand, hypothalamus-hippocampus circuitry emerges as an important neural pathway to control various activities, for example, behavioral impulsivity and stress response (Noble et al., 2019). To sum up, studying hypothalamic and/or hippocampal circuits separately or jointly by single-cell sequencing is essential. Therefore, in addition to hippocampal circuits discussed in the following chapter, we mainly summarize some transcriptomic studies about hypothalamic neurons and circuits by single-cell sequencing here, which are also discussed in Table 2.

In the functional sense, hypothalamic circuits are mainly responsible for maintaining homeostatic challenges, as exemplified by immune responses to coronavirus disease 2019 (COVID-19) (Poon, 2020; Mussa et al., 2021). Hanchate et al. (2020) have found that numerous neuropeptides are expressed in upstream neurons isolated from the hypothalamus by using Connect-seq, which can generate a complex molecular map and further allow the molecular and genetic interrogation of how neuronal components control its function in neural circuits. Of interest, 39 neuropeptides are coexpressed with glutamate or GABA, indicating the potential mechanisms for their excitatory and inhibitory effects. In detail, *Avp* has been observed for *PRV+* neurons in four areas of the hypothalamus. By contrast, *Tac1* and *Npy* have been detected in *PRV+* neurons in only one hypothalamic area, respectively, i.e., the dorsomedial hypothalamic nucleus (DMH) for *Tac1* and the arcuate hypothalamic nucleus (ARC) for *Npy*. These findings together show the diversity of molecular signatures among different subtypes of hypothalamic neurons (Hanchate et al., 2020). Moreover, Chen et al. (2017) have comprehensively evaluated hypothalamic neuron diversity in mice utilizing Drop-seq and defined 34 novel clusters of neurons with distinct transcriptional signatures. The neuropeptide expression profile across different subtypes of hypothalamic neurons has also been checked, and *Crabp1*^+^ neurons in arcuate hypothalamic nucleus (ARH) and *Pax6*^+^ neurons in the zona incerta (ZI) have been identified, suggesting that there are still many uncharacterized neuron subpopulations in the hypothalamus (Chen et al., 2017). In addition, the expression pattern of several transcription factors, for example, *Foxb1*, *Npas1*, and *Lhx8* have displayed a neuron subtype-specific pattern, which is in line with their functions of promoting neuron differentiation as well as identity (Chen et al., 2017).

Besides, some single-cell transcriptomic studies have focused on the specified regions of the hypothalamus, such as the hypothalamic preoptic region and the ventral posterior hypothalamus. To this end, Moffitt et al. (2016) have applied Drop-seq and MERFISH in the hypothalamic preoptic region and identified ∼70 neuronal populations among ∼1 million cells, including 43 subpopulations for inhibitory neurons and 23 subpopulations for excitatory neurons, together outlining a high-resolution framework for mechanistic exploration of behavior circuits in the hypothalamus. Of interest, the combination of MERFISH with measurements of immediate early genes can help to define specific neuron subpopulations activated by specific social behaviors in different physiological states (Moffitt et al., 2018). Recently, Lewis et al. (2020) have particularly targeted oxytocin neurons through SMART-Seq2 in combination with molecular targeting approaches and revealed that some autism risk genes, such as *Calb1*, *Kcnmb4*, *Reln*, and *Cnr1*, are enriched in parvocellular oxytocin neurons in comparison to magnocellular oxytocin neurons in the context of parallel processing circuits. Mickelsen et al. (2020) have investigated novel neuronal cell types in the ventral posterior hypothalamus and defined 20 neuronal and 18 non-neuronal cell populations by analyzing more than 16,000 single cells, providing a resource for investigating the circuit-level mechanisms.

On the other hand, increasing interest in aging-associated cognitive declines have given rise to the compensation-related utilization of neural circuits hypothesis, drawing more attention to age-related signature changes of hypothalamic neurons in neural circuits (Furusawa and Emoto, 2021; Kang et al., 2022). Hajdarovic et al. (2022a) have performed snRNA-seq of 40,064 hypothalamic nuclei obtained from young and aged female mice, respectively, and revealed an unexpected female-specific feature of hypothalamic aging. Of note, the master regulator of X-inactivation, Xist, has been found to be upregulated with age, especially in hypothalamic neurons, providing some correlative explanations for aging-related hypothalamic changes between neural circuits and behaviors (Hajdarovic et al., 2022a,b).

## Advances in studying other region neurons and neural circuits *via* single-cell sequencing

In the last chapter of this review, we would like to summarize some transcriptomic findings about neuronal diversity and neural circuit complexity in other regions in addition to the cerebral cortex and the hypothalamus, mainly involving hippocampus, subpallium, dorsal root ganglion (DRG), and ventral tegmental area (Li et al., 2016; Yu et al., 2021; Kamath et al., 2022), which are also included in Table 2. Moreover, representative single-cell transcriptomic studies in other central nervous system (CNS) regions, such as, retina, cerebellum, spinal cord, have been briefly summarized in this table as well. Yu et al. (2021) have identified interneuron diversity and complex interneuron lineages in the human subpallium using 10X Genomics. This study highlights the temporal and spatial specification of interneuron subpopulations, which could be linked to neurodevelopmental and psychiatric disorders due to neural circuit dysregulation (Sah et al., 2020; Burket et al., 2021). Importantly, they have selected some representative region-specific markers in the subpallium, for instance, NKX2-1 and LHX6 for the medial ganglionic eminence (MGE), MEIS2 and ZFHX3 for the lateral ganglionic eminence (LGE), and NR2F1 and NR2F2 for the caudal ganglionic eminence (CGE), giving some candidates used for cell classification (Yu et al., 2021).

As for the hippocampus, Ding et al. (2020) have identified the subiculum and the prosubiculum as two important regions of the mouse brain with differential transcriptomic cell types as well as hippocampal circuits. In the meanwhile, they have defined 27 types of transcriptomic cells in both two areas by applying both SMART-seq and 10X Genomics, where two kinds of molecularly and anatomically distinct circuits, i.e., subiculum and prosubiculum circuits, are centered, shedding light into historical findings (Cembrowski et al., 2018; Ding et al., 2020). Interestingly, some genes, such as *Col23a1*, *Id4*, *Rab3b*, *Abca8a*, *Unc5d*, *Lpl*, *Gpc3*, and *Car10*, associated with circuit formation, have shown a region-enriched expression pattern in the hippocampus-amygdaloid transition area, as highlighted in Figure 2 (Ding et al., 2020). Tosches et al. (2018) have investigated transcriptomic signatures of glutamatergic and GABAergic neurons in the hippocampus in reptiles, and revealed significant heterogeneity in the *ZBTB20+ ETV1+* cells. Recently, novel technologies to characterize new neurons in the adult hippocampus, including single-cell RNA sequencing, intravital imaging, etc., have been generalized by one review article (Denoth-Lippuner and Jessberger, 2021). More importantly, the characterization of hippocampal circuits would better explain the onset of several neurological and psychiatric diseases and further provide some promising therapeutic targets.

Dorsal root ganglion neurons, as an important type of sensory neurons, are involved in the generation, transmission, as well as regulation of different somatosensory signals. In turn, their dysfunction occurs in a variety of neuronal diseases (Nascimento et al., 2018; Meltzer et al., 2021; Balogh et al., 2022). Li et al. (2016) have categorized large and small diameter somatosensory neurons in the mouse DRG. Of special interest, large DRG neurons are classified into four types, and small diameter DRG neurons are categorized into six types, together providing a new classification system for understanding somatosensory neuron subtypes in sensory circuits (Li et al., 2016). Meanwhile, they have revealed 1745 DEGs from 197 neurons for principal component analysis. Single-cell real-time PCR have further verified the differential expression of marker genes in C1 (*n* = 6), C2 (*n* = 6), C3 (*n* = 6), C4 (*n* = 3), and C5 (*n* = 6) neurons (Li et al., 2016). At last, Kamath et al. (2022) have profiled 22,048 dopamine neurons in the context of Parkinson’s disease through snRNA-seq analysis, and addressed the importance of neuronal vulnerability in disease-associated degeneration, as exemplified by a representative single subtype with the expression of AGTR1. Usoskin et al. (2015) have revealed eleven subtypes of sensory neurons with markedly different molecular and operational properties using single-cell RNA sequencing. In a similar vein, Chiu et al. (2014) have applied the same procedure to identify three populations and six distinct subgroups from 334 single DRG sensory neurons. These findings together reveal the complexity and diversity of these new subsets of neurons underlying somatosensation.

To sum up, characterization of diverse neuron subpopulations in specific brain regions through single-cell sequencing facilitates the accurate identification of region- and/or disease-specific circuits. The correlation among various functional activities induced by similar or distinct neural circuits beyond regions will be better deciphered. Of special note, to figure out how various cortical circuits are related to distinct functional behaviors, is essential to categorize cortical neurons into subtypes, and study their disparate roles in the context of certain neural circuits. Furthermore, the combinative application of multiple sc/snRNA-seq techniques will validate these preliminary findings and give more robust indications for further mechanistic investigation.

## Concluding remarks

To date, single-cell sequencing has been widely applied in identifying neuronal diversity and neural circuit complexity across different brain regions and among various species. Technical advances in sc/snRNA-seq combined with spatial transcriptomics have enabled it to provide comprehensive anatomical and functional information with spatial localization messages and extend our conventional understanding of how region-specific neural circuits work and abnormal neural circuits contribute to neurological disorders. With the identification of new neuron subpopulations in different brain regions and the characterization of these region-specific circuits, enormous information obtained by single-cell sequencing will be translated into organized and systematical knowledge about brain cell ontogeny and function. Moreover, it is worth noting that most current studies have shown cell atlases of normal brain tissues. By contrast, the profile characterization in pathological conditions remains poorly understood, which will be an interesting direction in the future. Overall, understanding nervous system organization beyond the level of individual neurons supports deciphering specific molecular mechanisms of neuropsychiatric diseases.

## Author contributions

YX and LL conceived and designed the contents and layout of the mini-review with help from CZ. YX and LL wrote the first draft of the manuscript and CZ edited it. All authors read and approved the final manuscript.
